# Deep Induction: Induction Rules for (Truly) Nested Types

**DOI:** 10.1007/978-3-030-45231-5_18

**Published:** 2020-04-17

**Authors:** Patricia Johann, Andrew Polonsky

**Affiliations:** 8 ENS/CNRS, Université Paris-Saclay, Cachan, France; 9 University of Duisburg-Essen, Duisburg, Germany; Appalachian State University, Boone, NC USA

## Abstract

This paper introduces *deep induction*, and shows that it is the notion of induction most appropriate to nested types and other data types defined over, or mutually recursively with, (other) such types. Standard induction rules induct over only the top-level structure of data, leaving any data internal to the top-level structure untouched. By contrast, deep induction rules induct over *all* of the structured data present. We give a grammar generating a robust class of nested types (and thus ADTs), and develop a fundamental theory of deep induction for them using their recently defined semantics as fixed points of accessible functors on locally presentable categories. We then use our theory to derive deep induction rules for some common ADTs and nested types, and show how these rules specialize to give the standard structural induction rules for these types. We also show how deep induction specializes to solve the long-standing problem of deriving principled and practically useful structural induction rules for bushes and other *truly* nested types. Overall, deep induction opens the way to making induction principles appropriate to richly structured data types available in programming languages and proof assistants. Agda implementations of our development and examples, including two extended case studies, are available.

### 1 Introduction

This paper is concerned with the problem of inductive reasoning about inductive data types that are defined over, or are defined mutually recursively with, (other) such data types. Examples of such *deep data types* include, trivially, ordinary algebraic data types (ADTs), such as list and tree types; data types, such as the forest type, whose recursive occurrences appear below other type constructors; simple nested types, such as the type of perfect trees, whose recursive occurrences never appear below their own type constructors; truly[1] nested types, such as the type of bushes (also called *bootstrapped heaps* by Okasaki [16]), whose recursive occurrences do appear below their own type constructors; and GADTs. Proof assistants, including Coq and Agda, currently provide insufficient support for performing induction over deep data types. The induction rules, if any, they generate for such types induct over only their top-level structures, leaving any data internal to the top-level structure untouched. This paper develops a principle that, by contrast, inducts over *all* of the structured data present. We call this principle *deep induction*. Deep induction not only provides general support for solving problems that previously had only (usually quite painful and) *ad hoc* solutions, but also opens the way for incorporating automatic generation of useful induction rules for deep data types into proof assistants.

[1] Nested types that are defined over themselves are known as *truly nested types*.

To illustrate the difference between structural induction and deep induction, note that the data inside a structure of type $\mathtt{List\,a} = \mathtt{Nil} \mid \mathtt{Cons\,a\,(List\,a)}$ is treated monolithically (i.e., ignored) by the structural induction rule for lists:

$$\forall (\mathsf{a} : \mathsf{Set})\,(\mathtt{P} : \mathtt{List\,a} \to \mathsf{Set}) \to \mathtt{P\,Nil} \to \\ (\forall\,(\mathtt{x} : \mathtt{a})\,(\mathtt{xs} : \mathtt{List\,a}) \to \mathtt{P\,xs} \to \mathtt{P\,(Cons\,x\,xs)}) \to \forall\,(\mathtt{xs} : \mathtt{List\,a}) \to \mathtt{P\,xs}$$

By contrast, the deep induction rule for lists traverses not just the outer list structure with a predicate `P`, but also each data element of that list with a custom predicate `Q`:

$$\forall\,(\mathsf{a} : \mathsf{Set})\,(\mathtt{P} : \mathtt{List\,a} \to \mathsf{Set})\,(\mathtt{Q} : \mathtt{a} \to \mathsf{Set}) \to \\ \mathtt{P\,Nil} \to (\forall(\mathtt{x} : \mathtt{a})\,(\mathtt{xs} : \mathtt{List\,a}) \to \mathtt{Q\,x} \to \mathtt{P\,xs} \to \mathtt{P\,(Cons\,x\,xs)}) \to \\ \forall(\mathtt{xs} : \mathtt{List\,a}) \to \mathtt{List}^{\wedge}\,\mathtt{Q\,xs} \to \mathtt{P\,xs}$$

Here, $\mathtt{List}^{\wedge}$ lifts its argument predicate `Q` on data of type `a` to a predicate on data of type `List a` asserting that `Q` holds for every element of its argument list. The structural induction rule for lists is, like that for any ADT, recovered by taking the custom predicate in the corresponding deep rule to be $\lambda\mathtt{x}.\,\mathtt{True}$.

A particular advantage of deep induction is that it obviates the need to reflect properties as data types. For example, although the set of primes cannot be defined by an ADT, the primeness predicate `Prime` on the ADT of natural numbers *can* be lifted to a predicate $\mathtt{List}^{\wedge}\,\mathtt{Prime}$ characterizing lists of primes. Properties can then be proved for lists of primes using the following deep induction rule:

$$\forall(\mathtt{P} : \mathtt{List\,Nat} \to \mathsf{Set}) \to \mathtt{P\,Nil} \to \\ (\forall(\mathtt{x} : \mathtt{Nat})\,(\mathtt{xs} : \mathtt{List\,Nat}) \to \mathtt{Prime\,x} \to \mathtt{P\,xs} \to \mathtt{P\,(Cons\,x\,xs)}) \to \\ \forall(\mathtt{xs} : \mathtt{List\,Nat}) \to \mathtt{List}^{\wedge}\,\mathtt{Prime\,xs} \to \mathtt{P\,xs}$$

As we'll see in Sections 3, 4, and 5, the extra flexibility afforded by lifting predicates like `Q` and `Prime` on data internal to a structure makes it possible to derive useful induction principles for more complex types, such as truly nested ones.

In each of the above examples, a predicate on the data is lifted to a predicate on the list. This is an example of lifting a predicate on a type in a *non-recursive* position of an ADT's definition to the entire ADT. However, the predicate to be lifted can also be on the type in a *recursive* position of a definition — i.e., on the ADT being defined itself — and this ADT can appear below another type constructor in the definition. This is exactly the situation for the ADT `Forest a`, which appears below the type constructor `List` in the definition

$$\mathtt{Forest\,a} = \mathtt{FEmpty} \mid \mathtt{FNode\,a\,(List\,(Forest\,a))}$$

The induction rule Coq generates for forests is

$$\forall\,(\mathsf{a} : \mathsf{Set})\,(\mathtt{P} : \mathtt{Forest\,a} \to \mathsf{Set}) \to \mathtt{P\,FEmpty} \to \\ (\forall\,(\mathtt{x} : \mathtt{a})\,(\mathtt{ts} : \mathtt{List\,(Forest\,a)}) \to \mathtt{P\,(FNode\,x\,ts)}) \to \forall\,(\mathtt{x} : \mathtt{Forest\,a}) \to \mathtt{P\,x}$$

However, this is neither the induction rule we intuitively expect, nor is it expressive enough to prove even basic properties of forests that ought to be amenable to inductive proof. The approach of [11,12] does give the expected rule[2]

[2] This is equivalent to the rule as classically stated in Coq/Isabelle/HOL.

$$\forall\,(\mathsf{a} : \mathsf{Set})\,(\mathtt{P} : \mathtt{Forest\,a} \to \mathsf{Set}) \to \mathtt{P\,FEmpty} \to (\forall\,(\mathtt{x} : \mathtt{a})\,(\mathtt{ts} : \mathtt{List\,(Forest\,a)}) \to (\forall\,(\mathtt{k} < \mathtt{length\,ts}) \to \mathtt{P\,(ts!!k)}) \to \mathtt{P\,(FNode\,x\,ts)}) \to \forall\,(\mathtt{x} : \mathtt{Forest\,a}) \to \mathtt{P\,x}$$

But to derive it, a technique based on list positions is used to propagate the predicate to be proved over the list of forests that is the second argument to the data constructor `FNode`. Unfortunately, this technique does not obviously extend to other deep data types, including the type of "generalized forests" introduced in Section 4.4 below, which combines smaller generalized forests into larger ones using a type constructor `f` potentially different from `List`. Nevertheless, replacing $\forall\,(\mathtt{k} < \mathtt{length\,ts}) \to \mathtt{P\,(ts!!k)}$ in the expected rule with $\mathtt{List}^\wedge\,\mathtt{P\,ts}$, which is equivalent, reveals that it is nothing more than the special case for $\mathtt{Q} = \lambda\mathtt{x}.\,\mathtt{True}$ of the following deep induction rule for `Forest a`:

$$\forall\,(\mathsf{a} : \mathsf{Set})\,(\mathtt{P} : \mathtt{Forest\,a} \to \mathsf{Set})\,(\mathtt{Q} : \mathtt{a} \to \mathsf{Set}) \to \mathtt{P\,FEmpty} \to (\forall\,(\mathtt{x} : \mathtt{a})\,(\mathtt{ts} : \mathtt{List\,(Forest\,a)}) \to \mathtt{Q\,x} \to \mathtt{List}^\wedge\,\mathtt{P\,ts} \to \mathtt{P\,(FNode\,x\,ts)}) \to \forall\,(\mathtt{x} : \mathtt{Forest\,a}) \to \mathtt{Forest}^\wedge\,\mathtt{Q\,x} \to \mathtt{P\,x}$$

When types, like `Forest a` and `List (Forest a)` above, are defined by mutual recursion, their (deep) induction rules are defined by mutual recursion as well. For example, the induction rules for the ADTs

```
data Expr  = Lit Nat | Add Expr Expr | If BExpr Expr Expr
data BExpr = BLit Bool | And BExpr BExpr | Not BExpr | Equal Expr Expr
```

of integer and boolean expressions are defined by mutual recursion as

$$\begin{aligned}
&\forall(\mathtt{P} : \mathtt{Expr} \to \mathsf{Set})\,(\mathtt{Q} : \mathtt{BExpr} \to \mathsf{Set}) \to \\
&\quad(\forall(\mathtt{n} : \mathtt{Nat}) \to \mathtt{P\,(Lit\,n)}) \to \\
&\quad(\forall(\mathtt{e1} : \mathtt{Expr})\,(\mathtt{e2} : \mathtt{Expr}) \to \mathtt{P\,e1} \to \mathtt{P\,e2} \to \mathtt{P\,(Add\,e1\,e2)}) \to \\
&\quad(\forall(\mathtt{b} : \mathtt{BExpr})\,(\mathtt{e1} : \mathtt{Expr})\,(\mathtt{e2} : \mathtt{Expr}) \to \mathtt{Q\,b} \to \mathtt{P\,e1} \to \mathtt{P\,e2} \to \mathtt{P\,(If\,b\,e1\,e2)}) \to \\
&\quad(\forall(\mathtt{b} : \mathtt{Bool}).\,\mathtt{Q\,(BLit\,b)}) \to \\
&\quad(\forall(\mathtt{b1} : \mathtt{BExpr})\,(\mathtt{b2} : \mathtt{BExpr}) \to \mathtt{Q\,b1} \to \mathtt{Q\,b2} \to \mathtt{Q\,(And\,b1\,b2)}) \to \\
&\quad(\forall(\mathtt{b} : \mathtt{BExpr}) \to \mathtt{Q\,b} \to \mathtt{Q\,(Not\,b)}) \to \\
&\quad(\forall(\mathtt{e1} : \mathtt{Expr})\,(\mathtt{e2} : \mathtt{Expr}) \to \mathtt{P\,e1} \to \mathtt{P\,e2} \to \mathtt{Q\,(Equal\,e1\,e2)}) \to \\
&\quad(\forall(\mathtt{e} : \mathtt{Expr}) \to \mathtt{P\,e}) \;\times\; (\forall(\mathtt{b} : \mathtt{BExpr}) \to \mathtt{Q\,b})
\end{aligned}$$

### 2 The Key Idea

As the examples of the previous section suggest, the key to deriving deep induction rules from (deep) data type declarations is to parameterize the induction rules not just over a predicate over the top-level data type being defined, but over predicates on the types of primitive data they contain as well. These additional predicates are then lifted to predicates on any internal structures containing these data, and the resulting predicates on these internal structures are lifted to predicates on any internal structures containing structures at the previous level, and so on, until the internal structures at all levels of the data type definition, including the top level, have been so processed. Satisfaction of a predicate by the data at one level of a structure is then conditioned upon satisfaction of the appropriate predicates by *all* of the data at the preceding level.

The above deep induction rules were all obtained using this technique. For example, the deep induction rule for lists is derived by first noting that structures of type `List a` contain only data of type `a`, so that only one additional predicate parameter, which we called `Q` above, is needed. Then, since the only data structure internal to the type `List a` is `List a` itself, `Q` need only be lifted to lists containing data of type `a`. This is exactly what `List^ Q` does. Finally, the deep induction rule for lists is obtained by parameterizing the standard one over not just `P` but also `Q`, adding the additional hypothesis `Q x` to its second antecedent, and adding the additional hypothesis `List^ Q xs` to its conclusion.

The deep induction rule for forests is similarly obtained from the Coq-generated rule by first parameterizing over an additional predicate `Q` on the type `a` of data stored in the forest, then lifting `P` to a predicate on lists containing data of type `Forest a` and `Q` to forests containing data of type `a`, and, finally, adding the additional hypotheses `Q x` and `List^ P ts` to its second antecedent and the additional hypothesis `Forest^ Q x` to its conclusion.

Predicate liftings such as `List^` and `Forest^` may either be supplied as primitives, or be generated automatically from the definitions of the types themselves, as described in Section 4. For container types, lifting a predicate amounts to traversing the container and applying the argument predicate pointwise.

Our technique for deriving deep induction rules for ADTs, as well as its generalization to nested types given in Section 3, is both made precise and rigorously justified in Section 4 using the results of [13]. This paper can thus be seen as a concrete application, in the specific category Fam, of the very general semantics developed in [13]; indeed, our induction rules are computed as the interpretations of the syntax for nested types in Fam. A general methodology is extracted in Section 5. The rest of this paper can be read either as "just" describing how to generate deep induction rules in practice, or as also proving that our technique for doing so is provably correct and general. Our Agda code is at [14].

### 3 Extending to Nested Types

Appropriately generalizing the basic technique of Section 2 derives deep induction rules, and therefore structural induction rules, for nested types, including truly nested types and other deep nested types. Nested types generalize ADTs by allowing elements at one instance of a data type to depend on data at other instances of the same type so that, in effect, the entire family of instances is constructed simultaneously. That is, rather than defining standalone *families of inductive types*, one for each choice of types to which type constructors like `List` and `Tree` are applied, the type constructors for nested types define *inductive families of types*. The structural induction rule for a nested type must therefore account for its changing type parameters by parameterizing over an appropriately polymorphic predicate, and appropriately instantiating that predicate's type argument at each application site. For example, the structural induction rule for the nested type

$$\texttt{PTree}\,\texttt{a} = \texttt{PLeaf}\,\texttt{a} \mid \texttt{PNode}\,(\texttt{PTree}\,(\texttt{a} \times \texttt{a}))$$

of perfect trees is

$$\begin{array}{l}\forall\,(\mathtt{P} : \forall\,(\mathsf{a} : \mathsf{Set}) \to \mathtt{PTree\,a} \to \mathsf{Set}) \to \\ \quad (\forall\,(\mathsf{a} : \mathsf{Set})\,(\mathtt{x} : \mathtt{a}) \to \mathtt{P\,a\,(PLeaf\,x)}) \to \\ \quad (\forall\,(\mathsf{a} : \mathsf{Set})\,(\mathtt{x} : \mathtt{PTree}\,(\mathtt{a} \times \mathtt{a})) \to \mathtt{P}\,(\mathtt{a} \times \mathtt{a})\,\mathtt{x} \to \mathtt{P\,a\,(PNode\,x)}) \to \\ \quad \forall\,(\mathsf{a} : \mathsf{Set})\,(\mathtt{x} : \mathtt{PTree\,a}) \to \mathtt{P\,a\,x}\end{array}$$

and the structural induction rule for the nested type

```
data Lam a where
    Var :: a → Lam a
    App :: Lam a → Lam a → Lam a
    Abs :: Lam (Maybe a) → Lam a
```

of de Bruijn encoded lambda terms [9] with variables of type $\mathtt{a}$ is

$$\begin{array}{l}\forall(\mathtt{P} : \forall(\mathsf{a} : \mathsf{Set}) \to \mathtt{Lam\,a} \to \mathsf{Set}) \to \\ \quad (\forall(\mathsf{a} : \mathsf{Set})\,(\mathtt{x} : \mathtt{a}) \to \mathtt{P\,a\,(Var\,x)}) \to \\ \quad (\forall(\mathsf{a} : \mathsf{Set})\,(\mathtt{x} : \mathtt{Lam\,a})\,(\mathtt{y} : \mathtt{Lam\,a}) \to \mathtt{P\,a\,x} \to \mathtt{P\,a\,y} \to \mathtt{P\,a\,(App\,x\,y)}) \to \\ \quad (\forall(\mathsf{a} : \mathsf{Set})\,(\mathtt{x} : \mathtt{Lam\,(Maybe\,a)}) \to \mathtt{P\,(Maybe\,a)\,x} \to \mathtt{P\,a\,(Abs\,x)}) \to \\ \quad \forall(\mathsf{a} : \mathsf{Set})\,(\mathtt{x} : \mathtt{Lam\,a}) \to \mathtt{P\,a\,x}\end{array}$$

Deep induction rules for nested types must similarly account for their type constructors' changing type parameters while also parameterizing over the additional predicate on the type of data they contain. Letting $\mathtt{Pair}^\wedge\,\mathtt{Q}$ be the lifting of a predicate $\mathtt{Q}$ on $\mathtt{a}$ to pairs of type $\mathtt{a} \times \mathtt{a}$, so that $\mathtt{Pair}^\wedge\,\mathtt{Q}\,(\mathtt{x},\mathtt{y}) = \mathtt{Q\,x} \times \mathtt{Q\,y}$, this gives the deep induction rule

$$\begin{array}{l}\forall\,(\mathtt{P} : \forall\,(\mathsf{a} : \mathsf{Set}) \to (\mathtt{a} \to \mathsf{Set}) \to \mathtt{PTree\,a} \to \mathsf{Set}) \to \\ \quad (\forall\,(\mathsf{a} : \mathsf{Set})\,(\mathtt{Q} : \mathtt{a} \to \mathsf{Set})\,(\mathtt{x} : \mathtt{a}) \to \mathtt{Q\,x} \to \mathtt{P\,a\,Q\,(PLeaf\,x)}) \to \\ \quad (\forall\,(\mathsf{a} : \mathsf{Set})\,(\mathtt{Q} : \mathtt{a} \to \mathsf{Set})\,(\mathtt{x} : \mathtt{PTree}\,(\mathtt{a} \times \mathtt{a})) \to \mathtt{P}\,(\mathtt{a} \times \mathtt{a})\,(\mathtt{Pair}^\wedge\,\mathtt{Q})\,\mathtt{x} \to \\ \qquad\qquad\qquad\qquad\qquad\qquad \mathtt{P\,a\,Q\,(PNode\,x)}) \to \\ \quad \forall\,(\mathsf{a} : \mathsf{Set})\,(\mathtt{Q} : \mathtt{a} \to \mathsf{Set})\,(\mathtt{x} : \mathtt{PTree\,a}) \to \mathtt{PTree}^\wedge\,\mathtt{Q\,x} \to \mathtt{P\,a\,Q\,x}\end{array}$$

for perfect trees, and the deep induction rule

$$\begin{array}{l}\forall(\mathtt{P} : \forall(\mathsf{a} : \mathsf{Set}) \to (\mathtt{a} \to \mathsf{Set}) \to \mathtt{Lam\,a} \to \mathsf{Set}) \to \\ \quad (\forall(\mathsf{a} : \mathsf{Set})\,(\mathtt{Q} : \mathtt{a} \to \mathsf{Set})\,(\mathtt{x} : \mathtt{a}) \to \mathtt{Q\,x} \to \mathtt{P\,a\,Q\,(Var\,x)}) \to \\ \quad (\forall(\mathsf{a} : \mathsf{Set})\,(\mathtt{Q} : \mathtt{a} \to \mathsf{Set})\,(\mathtt{x} : \mathtt{Lam\,a})\,(\mathtt{y} : \mathtt{Lam\,a}) \to \mathtt{P\,a\,Q\,x} \to \mathtt{P\,a\,Q\,y} \to \\ \qquad\qquad\qquad\qquad\qquad\qquad \mathtt{P\,a\,Q\,(App\,x\,y)}) \to \\ \quad (\forall(\mathsf{a} : \mathsf{Set})\,(\mathtt{Q} : \mathtt{a} \to \mathsf{Set})\,(\mathtt{x} : \mathtt{Lam\,(Maybe\,a)}) \to \mathtt{P\,(Maybe\,a)}\,(\mathtt{Maybe}^\wedge\,\mathtt{Q})\,\mathtt{x} \to \\ \qquad\qquad\qquad\qquad\qquad\qquad \mathtt{P\,a\,Q\,(Abs\,x)}) \to \\ \quad \forall(\mathsf{a} : \mathsf{Set})\,(\mathtt{Q} : \mathtt{a} \to \mathsf{Set})\,(\mathtt{x} : \mathtt{Lam\,a}) \to \mathtt{Lam}^\wedge\,\mathtt{Q\,x} \to \mathtt{P\,a\,Q\,x}\end{array}$$

for lambda terms. As usual, the structural induction rules for these types can be recovered by setting $\mathtt{Q} = \lambda\mathtt{x}.\,\mathtt{True}$ in their deep induction rules. Moreover, the basic technique described in Section 2 can be recovered from the more general one described in this section by noting that the type arguments to ADT data type constructors don't change, and that the internal predicate parameter to $\mathtt{P}$ can therefore be lifted to the outermost level of ADT induction rules.

We conclude this section by giving both structural and deep induction rules for the following truly nested type of bushes [8]:

$$\mathtt{Bush\,a} \;=\; \mathtt{BNil} \mid \mathtt{BCons\,a\,(Bush\,(Bush\,a))}$$

(Note that this type is not even definable in Agda.) Correct and useful structural induction rules for bushes and other truly nested types have long been elusive. One recent effort to derive such rules has been recorded in [10], but the approach taken there is more *ad hoc* than not, and generates induction rules for data types *related* to the nested types of interest rather than for the original nested types themselves. To treat bushes, for example, Fu and Selinger rewrite the type $\mathtt{Bush\,a}$ as $\mathtt{NBush\,(Succ\,Zero)\,a}$, where $\mathtt{NBush} = \mathtt{NTimes\,Bush}$ and

$$\begin{array}{ll}
\mathtt{NTimes} & :: \; (\mathsf{Set} \to \mathsf{Set}) \to \mathsf{Nat} \to \mathsf{Set} \to \mathsf{Set} \\
\mathtt{NTimes\,p\,Zero\,s} & = \mathtt{s} \\
\mathtt{NTimes\,p\,(Succ\,n)\,s} & = \mathtt{p\,(NTimes\,p\,n\,s)}
\end{array}$$

Their induction rule for bushes is then given in terms of these rewritten ones as

$$\begin{array}{l}
\forall\,(\mathtt{a} : \mathsf{Set})\,(\mathtt{P} : \forall\,(\mathtt{n} : \mathtt{Nat}) \to \mathtt{NBush\,n\,a} \to \mathsf{Set}) \to \\
\quad (\forall\,(\mathtt{x} : \mathtt{a}) \to \mathtt{P\,Zero\,x}) \to \\
\quad (\forall\,(\mathtt{n} : \mathtt{Nat}) \to \mathtt{P\,(Succ\,n)\,BNil}) \to \\
\quad (\forall\,(\mathtt{n} : \mathtt{Nat})\,(\mathtt{x} : \mathtt{NBush\,n\,a})\,(\mathtt{xs} : \mathtt{NBush\,(Succ\,(Succ\,n))\,a}) \to \\
\qquad\qquad \mathtt{P\,n\,x} \to \mathtt{P\,(Succ\,(Succ\,n))\,xs} \to \mathtt{P\,(Succ\,n)\,(BCons\,x\,xs)}) \to \\
\quad \forall\,(\mathtt{n} : \mathtt{Nat})\,(\mathtt{xs} : \mathtt{NBush\,n\,a}) \to \mathtt{P\,n\,xs}
\end{array}$$

This approach appears promising, but is not yet fully mature. The core difficulty is that, although Fu and Selinger "hint at how the construction ... can be generalized to arbitrary nested types" and "give an example of nested data type [sic] that is hopefully general enough to make it clear what one would do in the general case" in Section 5 of [10], they do not show how to derive their induction rules in a uniform and principled way even for the "reasonably arbitrary and general" nested types they consider. As a result, it is unclear what guarantees that the induction rules they derive are correct, *either* for the original nested types *or* for their rewritten versions, or whether the induction rules for the rewritten nested types are sufficiently expressive to prove all results about the original nested types that one would expect to be provable by induction. This latter point echoes the issue with Coq-derived induction rules for forests mentioned above, and has the unfortunate effect of forcing users to manually write induction (and other) rules for such types for use in that system [17].

Direct application of the general technique illustrated above and explicated in full in Section 4 below derives the following first-ever useful induction rule for bushes, respectively — a full 20 years after they were first introduced!

$$\begin{array}{l}
\forall(\mathtt{P} : \forall(\mathtt{a} : \mathsf{Set}) \to \mathtt{Bush\,a} \to \mathsf{Set}) \to \\
\quad (\forall(\mathtt{a} : \mathsf{Set}) \to \mathtt{P\,a\,BNil}) \to \\
\quad (\forall(\mathtt{a} : \mathsf{Set})\,(\mathtt{x} : \mathtt{a})\,(\mathtt{y} : \mathtt{Bush\,(Bush\,a)}) \to \mathtt{P\,(Bush\,a)\,y} \to \mathtt{P\,a\,(BCons\,x\,y)}) \to \\
\quad \forall(\mathtt{a} : \mathsf{Set})\,(\mathtt{x} : \mathtt{Bush\,a}) \to \mathtt{P\,a\,x}
\end{array}$$

In the next section we will see that this rule is derivable from the following more general one:

$$\begin{array}{l}\forall\,(\mathtt{P} : \forall\,(\mathtt{a} : \mathsf{Set}) \to (\mathtt{a} \to \mathsf{Set}) \to \mathtt{Bush\,a} \to \mathsf{Set}) \to \\ \quad (\forall\,(\mathtt{a} : \mathsf{Set})\,(\mathtt{Q} : \mathtt{a} \to \mathsf{Set}) \to \mathtt{P\,a\,Q\,Bnil}) \to \\ \quad (\forall\,(\mathtt{a} : \mathsf{Set})\,(\mathtt{Q} : \mathtt{a} \to \mathsf{Set})\,(\mathtt{x} : \mathtt{a})\,(\mathtt{y} : \mathtt{Bush}\,(\mathtt{Bush\,a})) \to \\ \qquad\qquad \mathtt{Q\,x} \to \mathtt{P}\,(\mathtt{Bush\,a})\,(\mathtt{P\,a\,Q})\,\mathtt{y} \to \mathtt{P\,a\,Q}\,(\mathtt{BCons\,x\,y})) \to \\ \quad \forall\,(\mathtt{a} : \mathsf{Set})\,(\mathtt{Q} : \mathtt{a} \to \mathsf{Set})\,(\mathtt{x} : \mathtt{Bush\,a}) \to \mathtt{Bush}^\wedge\,\mathtt{Q\,x} \to \mathtt{P\,a\,Q\,x}\end{array}$$

## 4 Theoretical Foundations

This section gives a grammar generating a robust class of nested types, including ADTs and truly nested types, and recaps the semantics given in [13] for them from which we derive their deep induction rules. This entire paper can thus be read as a practical application of the abstract results of [13].

### 4.1 Categorical Preliminaries

We write $a : \mathcal{A}$ if $\mathcal{A}$ is category and $a$ is an object of $\mathcal{A}$. We write $0_{\mathcal{A}}$ and $1_{\mathcal{A}}$ for the initial and terminal objects of $\mathcal{A}$, and $o_{\mathcal{A}}$ and $!_{\mathcal{A}}$ for the unique maps $o_{\mathcal{A}} : 0_{\mathcal{A}} \to \mathcal{A}$ and $!_{\mathcal{A}} : \mathcal{A} \to 1_{\mathcal{A}}$, respectively. If $\mathcal{A}$ is the category $\mathsf{Set}$ of sets and functions between them, we write 0 for $0_{\mathsf{Set}}$, i.e., for $\emptyset$, and 1 for any 1-element set, i.e., for $1_{\mathsf{Set}}$. If $a : \mathcal{A}$ we write $K_a$ for the constantly $a$-valued functor on $\mathcal{A}$. The category $\mathsf{Fam}$, which we will use to interpret predicates, is given by:

**Definition 1.** *The category* $\mathsf{Fam}$ *comprises the following:*

- ***Objects****: An object of* $\mathsf{Fam}$ *is a pair* $(A, P)$ *where* $A : \mathsf{Set}$ *and* $P : A \to \mathsf{Set}$.
- ***Morphisms****: A morphism* $f : (A, P) \to (A', P')$ *in* $\mathsf{Fam}$ *is a pair* $(\alpha, \beta)$, *where* $\alpha : A \to A'$ *and* $\beta : \Pi_{a:A}\, Pa \to P'(\alpha a)$.
- ***Identities****: The identity morphism* $\underline{id}_{(A,P)} : (A, P) \to (A, P)$ *in* $\mathsf{Fam}$ *is* $(id_A, \lambda a : A.\, id_{Pa})$.
- ***Composition****: If* $(\alpha, \beta) : (A, P) \to (A', P')$ *and* $(\alpha', \beta') : (A', P') \to (A'', P'')$, *then the composition* $(\alpha', \beta') \,\underline{\circ}\, (\alpha, \beta) : (A, P) \to (A'', P'')$ *in* $\mathsf{Fam}$ *is defined by* $(\alpha', \beta') \,\underline{\circ}\, (\alpha, \beta) = (\alpha' \circ \alpha, \lambda a : A.\, \beta'(\alpha a) \circ \beta a)$.

### 4.2 Syntax and Semantics of ADTs

If $\mathcal{V}$ is a countable set of type variables, $V \subseteq \mathcal{V}$ is finite, $\alpha \in \mathcal{V}$, and we write $V, \alpha$ for $V \cup \{\alpha\}$, then the following grammar generates (representations of) all standard *polynomial ADTs* over $V$, i.e., all ADTs defined over data of primitive types:

$$\mathcal{A}^V \;:=\; 0 \mid 1 \mid \alpha \in V \mid \mathcal{A}^V + \mathcal{A}^V \mid \mathcal{A}^V \times \mathcal{A}^V \mid \mu\alpha.\mathcal{A}^{V,\alpha}$$

The grammar $\mathcal{A} = \bigcup_V \mathcal{A}^V$ also generates (representations of) *deep ADTs*, i.e., ADTs defined not just over data of the primitive types, but over data of other ADTs as well. For example, it generates the representation $\mathit{List}\,\alpha := \mu\beta.\, 1+\alpha\times\beta$ of the type $\mathtt{List\,a}$, the representation $\mathit{Forest}\,\alpha := \mu\beta.\, 1+\alpha\times\mu\gamma.\, 1+\beta\times\gamma$ of the type $\mathtt{Forest\,a}$, and the representation $\mu\delta.\, 1+(\mu\beta.\, 1+\alpha\times\mu\gamma.\, 1+\beta\times\gamma)\times\delta$ of the type `List (Forest a)`. Using Bekič's Lemma, it can also generate (representations of) ADTs defined by mutual recursion such as $Expr := \mu\alpha.\, s(\alpha, \mu\beta.\, t(\alpha,\beta))$ and $BExpr := \mu\beta.\, t(Expr, \beta)$, where $s(\alpha,\beta) := Nat + \alpha \times \alpha + \beta \times \alpha \times \alpha$ and $t(\alpha,\beta) := Bool + \beta \times \beta + \beta + \alpha \times \alpha$ for the ADTs of integer and boolean expressions from Section 1. ADTs with more than one type argument can be handled by tupling them into one or, equivalently, by noting that such ADTs are generated by the extension $\mathcal{N}$ of the grammar $\mathcal{A}$ given in Section 4.4. We adopt the usual conventions regarding free and bound type variables for $\mathcal{A}$.

As usual, ADTs are interpreted relative to environments.

**Definition 2.** *A* set environment *$\sigma$ is a function from a finite subset $V$ of $\mathcal{V}$ to* $\mathsf{Set}$*. We write $\mathsf{Env}^{\mathsf{Set}}_V$ for the set of set environments whose domain is $V$. If $A \in \mathsf{Set}$, $\sigma \in \mathsf{Env}^{\mathsf{Set}}_V$, and $\alpha \notin V$, then $\sigma[\alpha := A]$ is the set environment with domain $V, \alpha$ that extends $\sigma$ by mapping $\alpha$ to $A$. We write $\sigma\alpha$ in place of $\sigma(\alpha)$ for the image of $\alpha$ under $\sigma$, and $[]$ for the set environment with domain $V = \emptyset$.*

It is well-known that the ADTs generated by the grammar $\mathcal{A}$ have initial algebra semantics in the category $\mathsf{Set}$. That is, each such ADT $\mu\alpha.\, E$ can be interpreted as the carrier $\mu F$ of the initial algebra for the polynomial endofunctor $F$ on $\mathsf{Set}$ that interprets its body $E$. In particular, the final clause of the next definition is well-defined.

**Definition 3.** *The interpretation function $[\![\cdot]\!]^{\mathsf{Set}} : \mathcal{A}^V \to \mathsf{Env}^{\mathsf{Set}}_V \to \mathsf{Set}$ is:*

$$\begin{aligned}
[\![0]\!]^{\mathsf{Set}}\sigma &= 0\\
[\![1]\!]^{\mathsf{Set}}\sigma &= 1\\
[\![\alpha]\!]^{\mathsf{Set}}\sigma &= \alpha\sigma\\
[\![E_1 + E_2]\!]^{\mathsf{Set}}\sigma &= [\![E_1]\!]^{\mathsf{Set}}\sigma + [\![E_2]\!]^{\mathsf{Set}}\sigma\\
[\![E_1 \times E_2]\!]^{\mathsf{Set}}\sigma &= [\![E_1]\!]^{\mathsf{Set}}\sigma \times [\![E_2]\!]^{\mathsf{Set}}\sigma\\
[\![\mu\alpha.\, E]\!]^{\mathsf{Set}}\sigma &= \mu(A \mapsto [\![E]\!]^{\mathsf{Set}}\sigma[\alpha := A])
\end{aligned}$$

Like $\mathsf{Set}$, the category $\mathsf{Fam}$ has sufficient structure to interpret ADTs generated by the grammar $\mathcal{A}$. In particular, it interprets bodies of polynomial ADTs.

**Definition 4.** *The category* $\mathsf{Fam}$ *supports the following constructions:*

– ***Initial object**: The initial object $\underline{0}$ of $\mathsf{Fam}$ is $(0, K_0 : 0 \to \mathsf{Set})$. For $(A,P) : \mathsf{Fam}$, $(o_A, \lambda x : 0.\, o_{P(o_A x)}) : \underline{0} \to (A,P)$ is the unique map from $\underline{0}$ to $(A,P)$.*
– ***Terminal object**: The terminal object $\underline{1}$ of $\mathsf{Fam}$ is $(1, K_1 : 1 \to \mathsf{Set})$, where $()$ is the unique element of the set $1$ and $K_1() = 1$. For $(A,P) : \mathsf{Fam}$, $(!_A, \lambda a : A.\, !_{Pa}) : (A,P) \to \underline{1}$ is the unique map from $(A,P)$ to $\underline{1}$.*
– ***Coproducts**: Given $(A,P), (A',P') : \mathsf{Fam}$, the coproduct $(A,P) \,\underline{+}\, (A',P') : \mathsf{Fam}$ is $(A + A', P + P')$, where $P + P' : A + A' \to \mathsf{Set}$ is just the usual coproduct of $P$ and $P'$ as functions. The associated injections $\underline{\mathsf{inL}} : (A,P) \to (A,P) \,\underline{+}\, (A',P')$ and $\underline{\mathsf{inR}} : (A',P') \to (A,P) \,\underline{+}\, (A',P')$ are given by $\underline{\mathsf{inL}} = (inL, \lambda a : A.\, id_{Pa})$ and $\underline{\mathsf{inR}} = (inR, \lambda a' : A'.\, id_{P'a'})$. The coproduct $(\alpha,\beta) \,\underline{+}\, (\alpha',\beta') : (A,P) \,\underline{+}\, (A',P') \to (B,Q)$ of morphisms $(\alpha,\beta) : (A,P) \to (B,Q)$*

*and* $(\alpha', \beta') : (A', P') \to (B, Q)$ *is* $(\alpha + \alpha', \delta)$*, where* $\delta : \Pi_{x \in A+A'}(P + P')x \to Q((\alpha + \alpha')x)$ *is defined by* $\delta(\mathsf{inL}\, a) = \beta a$ *and* $\delta(\mathsf{inR}\, a') = \beta' a'$*. As expected,* $((\alpha, \beta) \underline{+} (\alpha', \beta')) \underline{\circ}\, \underline{\mathsf{inL}} = (\alpha, \beta)$ *and* $((\alpha, \beta) \underline{+} (\alpha', \beta')) \underline{\circ}\, \underline{\mathsf{inR}} = (\alpha', \beta')$*.*

– ***Products****: Given* $(A, P), (A', P') : \mathsf{Fam}$*, the product* $(A, P) \underline{\times} (A', P') : \mathsf{Fam}$ *is* $(A \times A', \lambda(a, a') : A \times A'. Pa \times P'a')$*. The associated projections* $\underline{\pi_1} : (A, P) \underline{\times} (A', P') \to (A, P)$ *and* $\underline{\pi_2} : (A, P) \underline{\times} (A', P') \to (A', P')$ *are given by* $\underline{\pi_1} = (\pi_1, \lambda(a, a') : A \times A'. \pi_1)$ *and* $\underline{\pi_2} = (\pi_2, \lambda(a, a') : A \times A'. \pi_2)$*. The product* $(\alpha, \beta) \underline{\times} (\alpha', \beta') : (A, P) \to (B, Q) \underline{\times} (B', Q')$ *of morphisms* $(\alpha, \beta) : (A, P) \to (B, Q)$ *and* $(\alpha', \beta') : (A, P) \to (B', Q')$ *is* $(\lambda a : A.(\alpha a, \alpha' a), \lambda a : A. \lambda x : Pa. (\beta a x, \beta' a x))$*. As expected,* $\underline{\pi_1} \underline{\circ} ((\alpha, \beta) \underline{\times} (\alpha', \beta')) = (\alpha, \beta)$ *and* $\underline{\pi_2} \underline{\circ} ((\alpha, \beta) \underline{\times} (\alpha', \beta')) = (\alpha', \beta')$*.*

To interpret ADTs generated by $\mathcal{A}$ in $\mathsf{Fam}$ we also need to be able to interpret expressions of the form $\mu\alpha.E$. This we do by computing the least fixed point in $\mathsf{Fam}$ of the functor $G : \mathsf{Fam} \to \mathsf{Fam}$ interpreting $E$. It is natural to try to do this using the same technique in $\mathsf{Fam}$ that gives its $\mathsf{Set}$-interpretation, i.e., by iterating $G$ $\omega$-many times starting from the initial object $\underline{0}$ of $\mathsf{Fam}$. This gives the least fixed point $\underline{\mu}G$ of $G$ as the colimit $G^\omega \underline{0}$ in $\mathsf{Fam}$ of the sequence

$$\underline{0} \hookrightarrow G\underline{0} \hookrightarrow G^2\underline{0} \hookrightarrow \ldots \hookrightarrow G^n\underline{0} \hookrightarrow \ldots \qquad (*)$$

This approach is indeed viable, and is formally justified by [13]. There, it is shown that if $\lambda$ is a regular cardinal, $\mathcal{C}$ is a locally $\lambda$-presentable category, and $G : \mathcal{C} \to \mathcal{C}$ is a $\lambda$-accessible functor drawn from a particular class of functors that goes far beyond just first-order polynomial ones, then the least fixed point $\underline{\mu}G$ of $G$ exists in $\mathcal{C}$ and can be computed as the transfinite colimit $G^\lambda \underline{0}$ of the sequence $\underline{0} \hookrightarrow G\underline{0} \hookrightarrow G^2\underline{0} \hookrightarrow \ldots \hookrightarrow G^n\underline{0} \hookrightarrow \ldots \hookrightarrow G^\omega\underline{0} \hookrightarrow \ldots \hookrightarrow G^\alpha\underline{0} \hookrightarrow \ldots$ over all $\alpha < \lambda$. That the sequence (*) computes $\underline{\mu}G$ for all polynomial functors on $\mathsf{Fam}$ then follows by taking $\lambda$ to be $\omega$, noting that $\mathsf{Fam}$ is locally finitely presentable, and recalling that all such functors are $\omega$-accessible. That (*) further computes $\underline{\mu}G$ for *every* functor $G$ on $\mathsf{Fam}$ that interprets an expression generated by $\mathcal{A}$ now follows easily by structural induction. We record this as:

**Theorem 1.** *If* $G : \mathsf{Fam} \to \mathsf{Fam}$ *is a functor interpreting an expression (with a distinguished variable) generated by the grammar* $\mathcal{A}$*, then the least fixed point* $\underline{\mu}G$ *of* $G$ *(with respect to that variable) is* $G^\omega\underline{0}$*. Concretely, the colimit* $G^\omega\underline{0}$ *can be computed as* $\varinjlim_{n \in \mathbb{N}}(A_n, P_n) = (A, P)$*, where* $A = \varinjlim_{n \in \mathbb{N}} A_n$ *with mediating morphisms* $\alpha_n : A_n \to A$*, and* $P$ *is defined by* $P\,x = \varinjlim_{n \in \mathbb{N}, y \in \alpha_n^{-1}(x)} P_n\, y$*.*

To define interpretations in $\mathsf{Fam}$ for ADTs generated by $\mathcal{A}$ we need the following analogue of Definition 2:

**Definition 5.** *A* predicate environment $\rho$ *is a function from a finite subset* $V$ *of* $\mathcal{V}$ *to* $\mathsf{Fam}$*. We write* $\mathsf{Env}_V^{\mathsf{Fam}}$ *for the set of predicate environments whose domain is* $V$*. If* $(A, P) \in \mathsf{Fam}$*,* $\rho \in \mathsf{Env}_V^{\mathsf{Fam}}$*, and* $\alpha \notin V$*, we write* $\rho[\alpha := (A, P)]$ *for the predicate environment with domain* $V, \alpha$ *that extends* $\rho$ *by mapping* $\alpha$ *to* $(A, P)$*. We write* $\alpha\rho$ *in place of* $\rho(\alpha)$ *for the image of* $\alpha$ *under* $\rho$*.*

*Let* $\sigma \in \mathsf{Env}_V^{\mathsf{Set}}$*. If* $\rho \in \mathsf{Env}_V^{\mathsf{Fam}}$ *is such that* $\pi_1(\alpha\rho) = \alpha\sigma$ *for all* $\alpha \in V$ *then we say that* $\rho$ *is a* lifting *of* $\sigma$*. We write* $\overline{\sigma}$ *for the particular lifting* $\rho$ *of* $\sigma$ *such*

*that* $\alpha\rho = (\alpha\sigma, K_1)$ *for all* $\alpha \in V$. *In addition, if* $\rho \in \mathsf{Env}_V^{\mathsf{Fam}}$ *maps each* $\alpha \in V$ *to* $(A_\alpha, P_\alpha)$ *then we write* $\pi_1\rho$ *for the set environment with domain* $V$ *mapping each* $\alpha \in V$ *to* $A_\alpha$. *We write* $[]$ *for the unique environment with domain* $V = \emptyset$.

We then have the following $\mathsf{Fam}$-interpretations for ADTs generated by $\mathcal{A}$:

**Definition 6.** *The interpretation function* $[\![\cdot]\!]^{\mathsf{Fam}} : \mathcal{A}^V \to \mathsf{Env}_V^{\mathsf{Fam}} \to \mathsf{Fam}$ *is:*

$$\begin{aligned}
[\![0]\!]^{\mathsf{Fam}}\rho &= \underline{0} \\
[\![1]\!]^{\mathsf{Fam}}\rho &= \underline{1} \\
[\![\alpha]\!]^{\mathsf{Fam}}\rho &= \alpha\rho \\
[\![E_1 + E_2]\!]^{\mathsf{Fam}}\rho &= [\![E_1]\!]^{\mathsf{Fam}}\rho \,\underline{+}\, [\![E_2]\!]^{\mathsf{Fam}}\rho \\
[\![E_1 \times E_2]\!]^{\mathsf{Fam}}\rho &= [\![E_1]\!]^{\mathsf{Fam}}\rho \,\underline{\times}\, [\![E_2]\!]^{\mathsf{Fam}}\rho \\
[\![\mu\alpha.E]\!]^{\mathsf{Fam}}\rho &= \underline{\mu}(Z \mapsto [\![E]\!]^{\mathsf{Fam}}\rho[\alpha := Z])
\end{aligned}$$

Before showing how to derive induction rules for the ADTs generated by $\mathcal{A}$ we prove two crucial lemmas linking their $\mathsf{Set}$- and $\mathsf{Fam}$-interpretations.

**Lemma 1.** *If* $E \in \mathcal{A}^V$ *and* $\rho \in \mathsf{Env}_V^{\mathsf{Fam}}$, *then* $\pi_1([\![E]\!]^{\mathsf{Fam}}\rho) = [\![E]\!]^{\mathsf{Set}}(\pi_1\rho)$. *Furthermore, if* $\pi_2(\beta\rho) = K_1$ *for all* $\beta \in V$, *then* $\pi_2([\![E]\!]^{\mathsf{Fam}}\rho) = K_1$.

*Proof.* By induction on the structure of expressions. The only non-trivial case is for $\mu\alpha.E \in \mathcal{A}^V$. Let $\rho \in \mathsf{Env}_V^{\mathsf{Fam}}$ be given. Letting $F : \mathsf{Set} \to \mathsf{Set}$ be defined by $FA = [\![E]\!]^{\mathsf{Set}}(\pi_1\rho)[\alpha := A]$ and $G : \mathsf{Fam} \to \mathsf{Fam}$ be defined by $G(A,Q) = [\![E]\!]^{\mathsf{Fam}}\rho[\alpha := (A,Q)]$, the induction hypothesis gives

$$\pi_1(G(A,Q)) = \pi_1([\![E]\!]^{\mathsf{Fam}}\rho[\alpha := (A,Q)]) = [\![E]\!]^{\mathsf{Set}}(\pi_1\rho)[\alpha := A] = FA \qquad (\dagger)$$

and if $\pi_2(\beta\rho) = K_1$ for all $\beta \in V$ then, moreover, $\pi_2(G(A,K_1)) = K_1$. We then have $\pi_1([\![\mu\alpha.E]\!]^{\mathsf{Fam}}\rho) = \pi_1(\underline{\mu}((A,Q) \mapsto [\![E]\!]^{\mathsf{Fam}}\rho[\alpha := (A,Q)])) = \pi_1(\underline{\mu}G) = \pi_1(\varinjlim_{n\in\mathbb{N}} G^n\underline{0}) = \varinjlim_{n\in\mathbb{N}}\pi_1(G^n\underline{0}) = \varinjlim_{n\in\mathbb{N}} F^n 0 = \mu F = \mu(A \mapsto [\![E]\!]^{\mathsf{Set}}(\pi_1\rho)[\alpha := A]) = [\![\mu\alpha.E]\!]^{\mathsf{Set}}(\pi_1\rho)$. Here, the fourth equality is justified by Theorem 1, and the fifth is justified by (†) and induction on $n$. If $\pi_2(\beta\rho) = K_1$ for all $\beta \in V$ as well, then $\pi_2([\![\mu\alpha.E]\!]^{\mathsf{Fam}}\rho) = \pi_2(\underline{\mu}((A,Q) \mapsto [\![E]\!]^{\mathsf{Fam}}\rho[\alpha := (A,Q)])) = \pi_2(\underline{\mu}G) = \pi_2(\varinjlim_{n\in\mathbb{N}} G^n\underline{0}) = \pi_2(\varinjlim_{n\in\mathbb{N}}(F^n 0, K_1)) = \lambda x.\varinjlim_{n\in\mathbb{N}, y\in\alpha_n^{-1}x} K_1 y = K_1$. Here, the morphisms $\alpha_n : F^n 0 \to \mu F$ are the mediating morphisms for the colimit, as in Theorem 1, and the fourth equality is justified by the fact that $\pi_2(G(A,K_1)) = K_1$ and induction on $n$.

**Corollary 1.** *If* $E$ *is closed then* $[\![E]\!]^{\mathsf{Fam}}[] = ([\![E]\!]^{\mathsf{Set}}[], K_1)$.

**Lemma 2.** *If* $\sigma \in \mathsf{Env}_V^{\mathsf{Set}}$, *and if* $F : \mathsf{Set} \to \mathsf{Set}$ *and* $G : \mathsf{Fam} \to \mathsf{Fam}$ *are given by* $FA = [\![E]\!]^{\mathsf{Set}}\sigma[\alpha := A]$ *and* $G(A,Q) = [\![E]\!]^{\mathsf{Fam}}\overline{\sigma}[\alpha := (A,Q)]$, *then* $\underline{\mu}G = (\mu F, K_1)$.

*Proof.* We have $\underline{\mu}G = \underline{\mu}((A,Q) \mapsto [\![E]\!]^{\mathsf{Fam}}\overline{\sigma}[\alpha := (A,Q)]) = [\![\mu\alpha.E]\!]^{\mathsf{Fam}}\overline{\sigma} = ([\![\mu\alpha.E]\!]^{\mathsf{Set}}\sigma, K_1) = (\mu F, K_1)$, where the third equality holds by Lemma 1.

#### 4.3 Induction Rules for ADTs

To derive induction rules for the ADTs generated by $\mathcal{A}$, we first observe that, given an ADT $\mu\alpha.E \in \mathcal{A}^V$ and a set environment $\sigma \in \mathsf{Env}_V^{\mathsf{Set}}$ interpreting its free variables, the interpretation $[\![E]\!]^{\mathsf{Set}}\sigma$ defines a functor $F_\sigma A = [\![E]\!]^{\mathsf{Set}}\sigma[\alpha := A]$ such that $[\![\mu\alpha.E]\!]^{\mathsf{Set}}\sigma = \mu(A \mapsto [\![E]\!]^{\mathsf{Set}}\sigma[\alpha := A]) = \mu(A \mapsto F_\sigma A) = \mu F_\sigma$. We can therefore think of $F_\sigma$ as representing the data type constructor associated with the ADT. Thus, as argued in [11,12], the semantic induction rule for proving predicates over the $\sigma$-instance of the ADT $\mu\alpha.E$ has the form

$$\forall (P : \mu F_\sigma \to \mathsf{Set}).\, ??? \to \forall (x : \mu F_\sigma).\, Px$$

for some appropriate hypotheses ???. We can use the Fam-interpretation of $E$ to discover a semantic counterpart to the hypotheses ???. Reflecting the resulting semantic rule for the $\sigma$-instance of $\mu\alpha.E$ back into the programming language syntax will then derive induction rules for polynomial ADTs.

To deduce what ??? is, we first observe that the conclusion $\forall (x : \mu F_\sigma).\, Px$ of the induction rule for the $\sigma$-instance of $\mu\alpha.E$ is isomorphic to the type of the second component of a morphism in Fam from $(\mu F_\sigma, K_1)$ to $(\mu F_\sigma, P)$ whose first component is *id*. Lemma 1 suggests that if we can see $(\mu F_\sigma, K_1)$ as $\underline{\mu} G$ for some functor $G : \mathsf{Fam} \to \mathsf{Fam}$, then we can fold over a $G$-algebra on $(\mu F_\sigma, P)$ in Fam to get such a morphism, i.e., to inhabit the type that is the structural induction rule for the $\sigma$-instance of $\mu\alpha.E$. This will provide a proof $ind_{\mu\alpha.E,\overline{\sigma}}\, P$ that the property $P$ holds for all elements of the $\sigma$-instance of $\mu\alpha.E$.

To this end, let $\rho \in \mathsf{Env}_V^{\mathsf{Fam}}$ be any lifting of $\sigma$, and consider again the functor $\hat{F}_\rho(A, Q) = [\![E]\!]^{\mathsf{Fam}}\rho[\alpha := (A, Q)]$ on Fam given in Lemma 1 (there called $G$). An $\hat{F}_\rho$-algebra structure on $(\mu F_\sigma, P)$ is a morphism $(k', k) : \hat{F}_\rho(\mu F_\sigma, P) \to (\mu F_\sigma, P)$ in Fam. Then $\pi_1(\hat{F}_\rho(\mu F_\sigma, P)) = \pi_1([\![E]\!]^{\mathsf{Fam}}\rho[\alpha := (\mu F_\sigma, P)]) = (\pi_1([\![E]\!]^{\mathsf{Fam}}\rho))[\alpha := \mu F_\sigma] = [\![E]\!]^{\mathsf{Set}}\sigma[\alpha := \mu F_\sigma] = F_\sigma(\mu F_\sigma)$, with the third equality holding by Lemma 1. If we take $k' = in$, then $k : \forall (x : F_\sigma(\mu F_\sigma)).\, \pi_2([\![E]\!]^{\mathsf{Fam}}\rho[\alpha := (\mu F_\sigma, P)])x \to P(in\, x)$, so that

$$\begin{aligned}
ind_{\mu\alpha.E,\,\rho} \quad &: \ \forall (P : \mu F_\sigma \to \mathsf{Set}).\\
&\qquad (\forall (x : F_\sigma(\mu F_\sigma)).\, \pi_2([\![E]\!]^{\mathsf{Fam}}\rho[\alpha := (\mu F_\sigma, P)])x \to P(in\, x))\\
&\qquad\quad \to \forall (x : \mu F_\sigma).\, Px\\
ind_{\mu\alpha.E,\,\rho}\, P\, k\, x &= \pi_2\, (fold^{\mathsf{Fam}}_{\mu\alpha.E,\,\rho}\, (in, k))\, x\, ()
\end{aligned}$$

Here, $fold^{\mathsf{Fam}}_{\mu\alpha.\,E,\,\rho}(in, k)$ is the unique $\hat{F}_\rho$-algebra morphism from $\underline{in} : \hat{F}_\rho(\underline{\mu}\hat{F}_\rho) \to \underline{\mu}\hat{F}_\rho$ to $(in, k)$ in Fam.

Taking $\rho = \overline{\sigma}$ in the above development derives the expected structural induction rules for ADTs generated by $\mathcal{A}$. But this development is actually far more flexible, since the induction rule it derives is parameterized over an *arbitrary* lifting $\rho$ of the set environment $\sigma$, and later specialized to $\overline{\sigma}$ to obtain structural induction rules for ADTs. The non-specialized rule can therefore be used to prove properties of ADTs that are parameterized over non-trivial (i.e., non-$K_1$) predicates on the type parameters to the type constructors induced by those ADTs; these are precisely our deep induction rules for ADTs.

As expected, the conclusion of an ADT's deep induction rule will have an additional hypothesis involving the lifting of this predicate to that ADT. As we have seen, the ability to lift a predicate $Q$ on a set $A$ to a predicate $T_Q$ on $TA$, where $T$ is an ADT's type constructor, is therefore central to deep induction. Every type constructor for every ADT generated by the grammar $\mathcal{A}$ has such a lifting. Concretely, it is computed as the second component of the interpretation in $\mathsf{Fam}$ of that data type. For example, the lifting $List_Q : List\, A \to \mathsf{Set}$ is $\pi_2[\![\mu\beta.\, 1 + \alpha \times \beta]\!]^{\mathsf{Fam}}[\alpha := (A, Q)]$. This can be coded in Agda as

```
List^ : ∀ {a : Set} → (a → Set) → (List a → Set)
List^ Q Nil  =  ⊤
List^ Q (Cons x xs)  =  Q x × List^ Q xs
```

*Example 1.* The deep induction rule for lists can be computed as the type of $ind_{List\,\alpha,\,\rho}$ for the ADT $List\,\alpha := \mu\beta.\, 1 + \alpha \times \beta$ and the predicate environment $\rho = [\alpha := (A, Q)]$ for $(A, Q) \in \mathsf{Fam}$. Letting $FY = [\![1 + \alpha \times \beta]\!]^{\mathsf{Set}}(\pi_1\rho)[\beta := Y] = 1 + A \times Y$ with the obviously named injections, we have that $\mu F = List\, A$. This gives the deep induction rule

$$\begin{aligned} ind_{List\,\alpha,\rho} : \forall (P : \mu F \to \mathsf{Set}).\, \forall\, (Q : A \to \mathsf{Set}).\\ (\forall (x : F(\mu F)).\, \pi_2\, ([\![1 + \alpha \times \beta]\!]^{\mathsf{Fam}}[\alpha := (A, Q), \beta := (\mu F, P)])x \to\\ P(in\, x)) \to \forall (x : \mu F).\, List_Q\, x \to P\, x \end{aligned}$$

Simplifying $\pi_2$'s argument gives $(1, K_1) \underline{+} (A, Q) \underline{\times} (\mu F, P)$. Its predicate part, obtained by applying $\pi_2$, is $K_1 + (Q \times P)$, so the hypotheses for $ind_{List\,\alpha,\rho}$ are

$$\begin{aligned} &\forall (x : 1 + A \times List\, A).(K_1 + (Q \times P))x \to P(in\, x)\\ = &(\forall (x : 1).\, 1 \to P\, Nil) \times (\forall (y : A).\, \forall (ys : List\, A).\, Q\, y \to P\, ys \to P\, (Cons\, y\, ys))\\ = &P\, Nil \times (\forall (y : A).\, \forall (ys : List\, A).\, Q\, y \to P\, ys \to P\, (Cons\, y\, ys)) \end{aligned}$$

Reflecting back into syntax gives the deep induction rule from Section 1:

```
∀ (a : Set) (P : List a → Set) (Q : a → Set) →
      P Nil → (∀(y : a) (ys : List a) → Q y → P ys → P (Cons y ys)) →
      ∀(xs : List a) → List^ Q xs → P xs
```

Taking $Q = K_1$ gives the usual structural induction rule for lists from Section 1.

*Example 2.* Since `Forest a` and `List (Forest a)` are mutually recursively defined, the deep induction rule for forests is defined by mutual recursion with the deep induction rule for lists. It can be computed as the type of $ind_{Forest\,\alpha,\,\rho}$ for the ADT $Forest\,\alpha := \mu\beta.\, \alpha \times \mu\gamma.\, 1 + \beta \times \gamma$ using the same technique as in Example 1. This gives the (deep) induction rule for forests from Section 1.

*Example 3.* Exactly the same technique delivers the deep induction rules from Section 1 for the mutually recursive ADTs `Expr` and `BExpr` whose representations are given before Definition 2.

### 4.4 Syntax and Semantics of Nested Types

We can use the technique from Section 4.3 to derive induction rules for nested types as well, including truly nested types and other deep nested types. To do so we first need an extension of the grammar $\mathcal{A}$ that generates these types.

Since nested types generalize ADTs to allow elements of a nested type at one instance of a type to depend on data at other instances of that nested type, they are interpreted as least fixed points not of ordinary (first-order) functors on $\mathsf{Fam}$, as ADTs are, but rather as least fixed points of higher-order such functors. Moreover, since nested types can be parameterized over any number of type arguments, the (first-order) functors interpreting them can have correspondingly arbitrary arities. For each $k \geq 0$ we therefore assume a countable set $\mathcal{F}^k$ of *functor variables of arity* $k$, disjoint for distinct $k$. We use lower case Greek letters for functor variables, write $\varphi^k$ to indicate that $\varphi \in \mathcal{F}^k$, and say that $\varphi$ has *arity* $k$ in this case. Type variables are exactly functor variables of arity 0; we continue to write $\alpha$, $\beta$, etc., rather than $\alpha^0$, $\beta^0$, etc., for them. We write $\mathcal{F} = \bigcup_{k\geq 0} \mathcal{F}^k$. If $V \subseteq \mathcal{F}$ is finite and $\varphi \in \mathcal{F}^k$ for some $k$, write $V, \varphi$ for $V \cup \{\varphi\}$.

**Definition 7.** *For a finite set $V$ of $\mathcal{F}$, the set of* (truly) nested data types over *$V$ is generated by the following grammar:*

$$\mathcal{N}^V \;:=\; 0 \mid 1 \mid \varphi^k\overline{\mathcal{N}^V} \mid \mathcal{N}^V + \mathcal{N}^V \mid \mathcal{N}^V \times \mathcal{N}^V \mid (\mu\varphi^k.\lambda\alpha_1...\alpha_k.\,\mathcal{N}^{V,\alpha_1,\ldots,\alpha_k,\varphi})\overline{\mathcal{N}^V}$$

Here, $\varphi^k \in V$ and the lengths of the vectors of terms in $\overline{\mathcal{N}^V}$ in the third and final clauses of the above grammar are both $k$.

The grammar $\mathcal{N} = \bigcup_V \mathcal{N}^V$ generalizes $\mathcal{A}$ by allowing recursion not just at the level of type variables, but also at the level of functor variables. This reflects the fact that, in programming language syntax, nested types can be parameterized over both types and type constructors. For example, $\mathcal{N}^V$ generates the representation $\mathit{PTree}\,\alpha := \big(\mu\varphi^1.\lambda\beta.\beta + \varphi(\beta \times \beta)\big)\,\alpha \in \mathcal{N}^\alpha$ of the type $\mathtt{PTree\,a}$, the representation $\mathit{Lam}\,\alpha := \big(\mu\varphi^1.\lambda\beta.\beta + \varphi\beta \times \varphi\beta + \varphi(\beta{+}1)\big)\,\alpha \in \mathcal{N}^\alpha$ of the type $\mathtt{Lam\,a}$ and the representation $\mathit{Bush}\,\alpha := \big(\mu\varphi^1.\lambda\beta.\,1 + \beta \times \varphi\,(\varphi\,\beta)\big)\,\alpha \in \mathcal{N}^\alpha$ of the type $\mathtt{Bush\,a}$. But it also generates the representation $\mathit{GForest}\,\varphi\,\alpha := \mu\beta.\,1 + \alpha \times \varphi\,\beta \in \mathcal{N}^{\varphi,\alpha}$ of the following nested type of generalized forests with data of type $\mathtt{a}$:

$$\mathtt{GForest\,f\,a} \;=\; \mathtt{FEmpty} \mid \mathtt{FNode\,a\,(f\,(GForest\,f\,a))}$$

This type is higher-order in the sense that the type constructor `GForest` takes not just a type, but also a (unary) type constructor, as an argument. It therefore cannot be expressed as an element of $\mathcal{A}$, and thus demonstrates the benefit of working with the more expressive grammar $\mathcal{N}$. On the other hand, it is decidedly ADT-like, in the sense that it defines a family of inductive types rather than an inductive family of types. In fact, if `f` were a type constructor induced by a nested type generated by our grammar, then `GForest f a` and `f (GForest f a)` would be mutually recursively defined. In this case, generalizing Example 2, their structural induction rules would also be defined by mutual recursion.

It is not hard to see that $\mathcal{A} \subseteq \mathcal{N}$. Moreover, the grammar $\mathcal{N}$ allows nested types to be parameterized over (other) nested data types, just as $\mathcal{A}$ allows ADTs to be parameterized over (other) ADTs. For instance, we could have perfect trees of lists or binary trees, bushes of perfect trees, etc.

We have the following notions of functor and application in $\mathsf{Fam}$:

**Definition 8.** *A* ($k$-ary) lifted functor $G : \mathsf{Fam}^k \to \mathsf{Fam}$ *is a pair* $(F, P)$, *where* $F : \mathsf{Set}^k \to \mathsf{Set}$ *and* $P : \forall (X_1, P_1)....(X_k, P_k).\, F X_1...X_k \to \mathsf{Set}$ *is a* $\mathsf{Fam}$*-indexed predicate. The* application *of a functor* $(F, P) : \mathsf{Fam}^k \to \mathsf{Fam}$ *to an object* $(A_1, Q_1), ...., (A_k, Q_k)$ *of* $\mathsf{Fam}^k$ *is given by*

$$(F, P)(A_1, Q_1)...(A_k, Q_k) \;=\; (F A_1...A_k,\; P(A_1, Q_1)...(A_k, Q_k))$$

We call a lifted functor $G = (F, P)$ a *lifting* of $F$ from $\mathsf{Set}$ to $\mathsf{Fam}$, and call $P$ a $\mathsf{Fam}$*-indexed predicate.* A $\mathsf{Set}$*-indexed predicate* is a $\mathsf{Fam}$-indexed predicate that does not depend on its arguments' second components. We extend the notions of *set environment* and *predicate environment* from Definitions 2 and 5 as follows:

**Definition 9.** *A* set environment $\sigma$ *is a mapping from a finite subset* $V = \{\varphi_1^{k_1}, ..., \varphi_n^{k_n}\}$ *of* $\mathcal{F}$ *such that* $\varphi_i \sigma : \mathsf{Set}^{k_i} \to \mathsf{Set}$ *for* $i = 1, ..., n$. *We write* $\mathsf{Env}_V^{\mathsf{Set}}$ *for the set of set environments whose domain is* $V$. *If* $F \in \mathsf{Set}^k \to \mathsf{Set}$, $\sigma \in \mathsf{Env}_V^{\mathsf{Set}}$, *and* $\varphi^k \notin V$, *we write* $\sigma[\varphi := F]$ *for the set environment with domain* $V, \varphi$ *that extends* $\sigma$ *by mapping* $\varphi$ *to* $F$. *Similarly, a* predicate environment $\rho$ *is a mapping from a finite subset* $V = \{\varphi_1^{k_1}, ..., \varphi_n^{k_n}\}$ *of* $\mathcal{F}$ *such that* $\varphi_i \rho : \mathsf{Fam}^{k_i} \to \mathsf{Fam}$ *is a lifted functor for* $i = 1, ..., n$. *We write* $\mathsf{Env}_V^{\mathsf{Fam}}$ *for the set of predicate environments whose domain is* $V$. *If* $(F, P) \in \mathsf{Fam}^k \to \mathsf{Fam}$, $\rho \in \mathsf{Env}_V^{\mathsf{Fam}}$, *and* $\varphi^k \notin V$, *we write* $\rho[\varphi := (F, P)]$ *for the predicate environment with domain* $V, \varphi$ *that extends* $\rho$ *by mapping* $\varphi$ *to* $(F, P)$.

The notions of a predicate environment being a lifting of a set environment and the notations $\overline{\sigma}$, $\pi_1 \rho$, and $[]$ are now extended in the obvious ways.

The following interpretations of nested types generated by $\mathcal{N}$ in the locally finitely presentable categories $\mathsf{Set}$ and $\mathsf{Fam}$ are shown in [13] to be well-defined:

**Definition 10.** *The interpretation functions* $[\![\cdot]\!]^{\mathsf{Set}} : \mathcal{N}^V \to \mathsf{Env}_V^{\mathsf{Set}} \to \mathsf{Set}$ *and* $[\![\cdot]\!]^{\mathsf{Fam}} : \mathcal{N}^V \to \mathsf{Env}_V^{\mathsf{Fam}} \to \mathsf{Fam}$ *are:*

$$\begin{aligned}
[\![0]\!]^{\mathsf{Set}}\sigma &= 0\\
[\![1]\!]^{\mathsf{Set}}\sigma &= 1\\
[\![\varphi^k E_1...E_k]\!]^{\mathsf{Set}}\sigma &= (\varphi\sigma)\overline{([\![E_i]\!]^{\mathsf{Set}}\sigma)}\\
[\![E_1 + E_2]\!]^{\mathsf{Set}}\sigma &= [\![E_1]\!]^{\mathsf{Set}}\sigma + [\![E_2]\!]^{\mathsf{Set}}\sigma\\
[\![E_1 \times E_2]\!]^{\mathsf{Set}}\sigma &= [\![E_1]\!]^{\mathsf{Set}}\sigma \times [\![E_2]\!]^{\mathsf{Set}}\sigma\\
[\![(\mu\varphi^k.\lambda\alpha_1...\alpha_k.\,E)E_1...E_k]\!]^{\mathsf{Set}}\sigma &= (\mu(F \mapsto \lambda A_1...A_k.\\
&\qquad [\![E]\!]^{\mathsf{Set}}\sigma\overline{[\alpha_i := A_i]}[\varphi := F]))\overline{([\![E_i]\!]^{\mathsf{Set}}\sigma)}
\end{aligned}$$

$$
\begin{aligned}
[\![0]\!]^{\mathsf{Fam}}\rho &= \underline{0}\\
[\![1]\!]^{\mathsf{Fam}}\rho &= \underline{1}\\
[\![\varphi^k E_1...E_k]\!]^{\mathsf{Fam}}\rho &= (\varphi\rho)\overline{([\![E_i]\!]^{\mathsf{Fam}}\rho)}\\
[\![E_1 + E_2]\!]^{\mathsf{Fam}}\rho &= [\![E_1]\!]^{\mathsf{Fam}}\rho \,\underline{+}\, [\![E_2]\!]^{\mathsf{Fam}}\rho\\
[\![E_1 \times E_2]\!]^{\mathsf{Fam}}\rho &= [\![E_1]\!]^{\mathsf{Fam}}\rho \,\underline{\times}\, [\![E_2]\!]^{\mathsf{Fam}}\rho\\
[\![(\mu\varphi^k.\lambda\alpha_1...\alpha_k.\, E)E_1...E_k]\!]^{\mathsf{Fam}}\rho &= (\underline{\mu}(F \mapsto \lambda Z_1...Z_k.\\
&\qquad [\![E]\!]^{\mathsf{Fam}}\rho\overline{[\alpha_i := Z_i]}[\varphi := F]))\overline{([\![E_i]\!]^{\mathsf{Fam}}\rho)}
\end{aligned}
$$

#### 4.5 Induction Rules for Nested Types

Straightforward generalization of the analysis in Section 4.3 to $\mathcal{N}$ gives induction rules for the type constructors nested types induce. Given a nested type $(\mu\varphi^k.\lambda\alpha_1...\alpha_k.\, E)E_1...E_k \in \mathcal{N}^V$ with type constructor $T = \mu\varphi^k.\lambda\alpha_1...\alpha_k.\, E$ and a set environment $\sigma \in \mathsf{Env}_V^{\mathsf{Set}}$ interpreting its free variables, we have that

$$[\![T\overline{E_i}]\!]^{\mathsf{Set}}\sigma = \mu(F \mapsto \lambda A_1...A_k.\, [\![E]\!]^{\mathsf{Set}}\sigma\overline{[\alpha_i := A_i]}[\varphi := F])\overline{([\![E_i]\!]^{\mathsf{Set}}\sigma)} = (\mu H_\sigma)\overline{([\![E_i]\!]^{\mathsf{Set}}\sigma)}$$

where the higher-order functor $H_\sigma$ on $\mathsf{Set}$ is defined by

$$H_\sigma F A_1...A_k = [\![E]\!]^{\mathsf{Set}}\sigma\overline{[\alpha_i := A_i]}[\varphi := F]$$

For any lifting $\rho$ of $\sigma$, the predicate counterpart to $H_\sigma$ is the higher-order functor $\hat{H}_\rho$ on $\mathsf{Fam}$ whose action on a $k$-ary lifted functor $(F, P)$ is the $k$-ary lifted functor $\hat{H}_\rho(F, P)$ given by

$$\hat{H}_\rho\,(F, P)\,(A_1, Q_1)....(A_k, Q_k) \;=\; [\![E]\!]^{\mathsf{Fam}}\rho\overline{[\alpha := (A_i, Q_i)]}[\varphi := (F, P)]$$

The induction rule $ind_{T,\,\rho}$ for proving predicates over the $\sigma$-instance of the type constructor $T$ relative to the lifting $\rho$ is thus given by

$$
\begin{aligned}
ind_{T,\,\rho} :\;\; & \forall(P : \forall\overline{(A_i, Q_i)}.(\mu H_\sigma)\,\overline{A_i} \to \mathsf{Set}).\\
&\quad (\forall\overline{(A_i, Q_i)}.\, \pi_2(\hat{H}_\rho(\mu H_\sigma, P))\overline{(A_i, Q_i)} \to P\overline{(A_i, Q_i)}) \to\\
&\quad (\forall\overline{(A_i, Q_i)}.\, \pi_2(\mu\hat{H}_\rho)\overline{(A_i, Q_i)} \to P\overline{(A_i, Q_i)})\\
=\;& \forall(P : \forall\overline{(A_i.Q_i)}.(\mu H_\sigma)\,\overline{A_i} \to \mathsf{Set}).\\
&\quad (\forall\overline{(A_i, Q_i)}.\, \forall(x : H_\sigma(\mu H_\sigma)\overline{A_i}).\\
&\qquad \pi_2(\hat{H}_\rho(\mu H_\sigma, P))\overline{(A_i, Q_i)}\, x \to P\overline{(A_i, Q_i)}(in\, x)) \to\\
&\quad (\forall\overline{(A_i, Q_i)}.\, \forall(x : (\mu H_\sigma)\overline{A_i}).\, \pi_2(\mu\hat{H}_\rho)\overline{(A_i, Q_i)}\, x \to P\overline{(A_i, Q_i)}x)\\
ind_{T,\,\rho} =\;& \lambda\; P\, k\, \overline{(A_i, Q_i)}.\, \pi_2(fold^{\mathsf{Fam}}_{T,\,\rho}\,(in, k))
\end{aligned}
$$

To get analogues for nested types of the structural induction rules for ADTs note that, since each $\sigma$-instance of the type constructor $T = \mu\varphi^k.\lambda\alpha_1...\alpha_k.\, E$ associated with a nested type $(\mu\varphi^k.\lambda\alpha_1...\alpha_k.E)E_1...E_k \in \mathcal{N}^V$ gives rise to an inductive family of types, the appropriate notion of predicate for a nested type is actually a $\mathsf{Set}$-indexed predicate. By direct analogy with structural induction for ADTs, the structural induction rule for a nested type with type constructor $T$ whose $\sigma$-instance is interpreted by $\mu H_\sigma$ is then

$$\begin{aligned}
&\forall(P : \forall\overline{A_i}.(\mu H_\sigma)\,\overline{A_i} \to \mathsf{Set}).\\
&\quad(\forall\overline{A_i}.\,\forall(x : H_\sigma(\mu H_\sigma)\overline{A_i}).\,\pi_2(\hat{H}_{\overline{\sigma}}(\mu H_\sigma, \hat{P}))\overline{(A_i, K_1)}\,x \to \hat{P}\overline{(A_i, K_1)}(in\,x)) \to\\
&\quad(\forall\overline{A_i}.\,\forall(x : (\mu H_\sigma)\overline{A_i}).\,\pi_2(\mu\hat{H}_{\overline{\sigma}})\overline{(A_i, K_1)}\,x \to \hat{P}\overline{(A_i, K_1)}x)\\
=\;&\forall(P : \forall\overline{A_i}.(\mu H_\sigma)\,\overline{A_i} \to \mathsf{Set}).\\
&\quad(\forall\overline{A_i}.\forall(x : H_\sigma(\mu H_\sigma)\overline{A_i}).\,\pi_2(\hat{H}_{\overline{\sigma}}(\mu H_\sigma, \hat{P}))\overline{(A_i, K_1)}\,x \to \hat{P}\overline{(A_i, K_1)}(in\,x)) \to\\
&\quad(\forall\overline{A_i}.\,\forall(x : (\mu H_\sigma)\overline{A_i}).\,P\overline{A_i}x)
\end{aligned} \tag{‡}$$

where $\hat{P}$ is defined below. To see that the structural induction rule (‡) is indeed a specialization of $ind_{T,\,\rho}$, suppose we are given a predicate $P : \forall\overline{(A_i, Q_i)}.\,(\mu H_\sigma)\overline{A_i} \to \mathsf{Set}$ for a nested type with type constructor $T$ whose $\sigma$-instance is interpreted by $\mu H_\sigma$, together with induction hypotheses

$$R \;=\; \forall\overline{A_i}.\forall(x : H_\sigma(\mu H_\sigma)\overline{A_i}).\,\pi_2(\hat{H}_{\overline{\sigma}}(\mu H_\sigma, \hat{P}))\overline{(A_i, K_1)}\,x \to \hat{P}\overline{(A_i, K_1)}(in\,x)$$

Let $\hat{P} : \forall\overline{(A_i, Q_i)}.\,(\mu H_\sigma)\overline{A_i} \to \mathsf{Set}$ be the $\mathsf{Fam}$-indexed predicate $\hat{P} = \lambda\overline{(A_i, Q_i)}.\,P\overline{A_i}$, and consider the instantiation $ind_{T,\,\overline{\sigma}}\;\hat{P}\;\hat{R}$, where the induction hypothesis $\hat{R} \;:\; \forall\overline{(A_i, Q_i)}.\,\forall(x : H_\sigma(\mu H_\sigma)\overline{A_i}).\,\pi_2(\hat{H}_{\overline{\sigma}}(\mu H_\sigma, \hat{P}))\overline{(A_i, Q_i)}x \to \hat{P}\overline{(A_i, Q_i)}(in\,x)$ for $ind_{T,\,\overline{\sigma}}$ is given by $\hat{R}\,\overline{(A_i, Q_i)}\,x\,y \;=\; R\,\overline{A_i}\,x\,(\pi_2(\hat{H}_{\overline{\sigma}}(\mu H_\sigma, \hat{P})\,t)\,x\,y)$.

### 5 The General Methodology

We can distill from the foundations given in Section 4 a general methodology that will derive correct deep induction rules for any nested type generated by $\mathcal{N}$. Concretely, this methodology comprises the following steps:

1. Given a nested data type definition $D$, translate its type constructor into an expression $N$ in the grammar $\mathcal{N}$ (or, more simply, $\mathcal{A}$, if $D$ defines an ADT).
2. Interpret $N$ in $\mathsf{Set}$ to get a fixpoint equation defining $D$ as $\mu H$ for some (higher-order) operator $H$.
3. Reinterpret $N$ in $\mathsf{Fam}$ to define a corresponding (higher-order) operator $\hat{H}$ on predicates whose fixed point $\mu\hat{H}$ is an inductive predicate on $\mu H$, i.e., on $D$.
4. Initiality of $\mu\hat{H}$ guarantees that there is a unique predicate morphism from $\mu\hat{H}$ to any other predicate $P$ admitting an $\hat{H}$-algebra structure. This gives $D$'s deep induction rule.

These are precisely the steps carried out in all of our examples, including those below, which illustrate the derivation for nested types given in Section 4.5.

*Example 4.* Since the nested type $Lam\,\alpha := \big(\mu\varphi^1.\lambda\beta.\beta + \varphi\beta \times \varphi\beta + \varphi(\beta{+}1)\big)\,\alpha$ of lambda terms is uniform in its index $\alpha$, it induces a type constructor $Lam := \mu\varphi^1.\lambda\beta.\beta + \varphi\beta \times \varphi\beta + \varphi(\beta{+}1)$. Writing $H$ for $H_{[]}$ and $\hat{H}$ for $\hat{H}_{[]}$, and letting

$$H\,F\,A \;=\; [\![\beta + \varphi\beta \times \varphi\beta + \varphi(\beta + 1)]\!]^{\mathsf{Set}}[\beta := A][\varphi := F] \;=\; A + FA \times FA + F(A + 1)$$

we have that $\mu H = Lam$ and that the predicate counterpart $\hat{H}$ to $H$ is given by

$$\begin{aligned}\hat{H}\,(F,\hat{P})\,(A,Q) &= [\![\beta + \varphi\beta \times \varphi\beta + \varphi(\beta+1)]\!]^{\mathsf{Fam}}[\beta := (A,Q)][\varphi := (F,\hat{P})]\\ &= (A,Q)\,\underline{+}\,(F,\hat{P})(A,Q)\,\underline{\times}\,(F,\hat{P})(A,Q)\,\underline{+}\,(F,\hat{P})((A,Q)\,\underline{+}\,(1,K_1))\\ &= (A + FA \times FA + F(A+1),\\ &\qquad \pi_2((A,Q)\,\underline{+}\,(F,\hat{P})(A,Q)\,\underline{\times}\,(F,\hat{P})(A,Q)\,\underline{+}\,(F,\hat{P})((A,Q)\,\underline{+}\,(1,K_1)))\end{aligned}$$

Reflecting $\mu\hat{H}$ back into syntax gives the inductive predicate

```
Lam^ : ∀(a : Set) → (a → Set) → (Lam a → Set) where
  Var^ : ∀(a : Set) (Q : a → Set) (x : a) → Q x → Lam^ a Q (Var x)
  App^ : ∀(a : Set) (Q : a → Set) (x : Lam a) (y : Lam a) → Lam^ a Q x →
                                  Lam^ a Q y → Lam^ a Q (App x y)
  Abs^ : ∀(a : Set) (Q : a → Set) (x : Lam a) → Lam^ (Maybe a) (Maybe^ a Q) x →
                                  Lam^ a Q (Abs x)
```

Now, if $P$ is any other predicate on $Lam$ admitting an $\hat{H}$-algebra structure, then there must exist a morphism $k : \forall(x : A + Lam\,A \times Lam\,A + Lam(A+1)).\,(Q + P\,A\,Q \times P\,A\,Q + P(A+1)((+1)^\wedge\,Q))x \to P\,A\,Q\,(in\,x)$, i.e., $k = (k_1, k_2, k_3)$, where

$$\begin{aligned}&k_1 : \forall(x : A).\,Q\,x \to P\,A\,Q\,(Var\,x)\\ &k_2 : \forall(x : Lam\,A).\,\forall(y : Lam\,A).\,P\,A\,Q\,x \to P\,A\,Q\,y \to P\,A\,Q\,(App\,x\,y)\\ &k_3 : \forall(x : Lam\,(A+1)).\,P\,(A+1)\,((+1)^\wedge\,Q)\,x \to P\,A\,Q\,(Abs\,x)\end{aligned}$$

Since `Lam^` reflects the initial $\hat{H}$-algebra, there is a unique algebra morphism from $in : \hat{H}(\mu\hat{H}) \to \mu\hat{H}$ to the $\hat{H}$-algebra $k$ on $P$, i.e., from $\mu\hat{H}$ to $P$. Reflecting this morphism back into syntax gives the deep induction rule for lambda terms from Section 3.

The deep induction rule for lambda terms can be used to prove, e.g., properties of lambda terms whose variables are represented by prime numbers or lambda terms over strings that can represent variable names. It can also be used to prove properties of lambda terms over lambda terms, such as the associativity laws needed to show that the functor $Lam$ is a monad; such a proof is included as the first case study in the accompanying Agda code. The second uses deep induction rule we derive in Example 5 to prove some results about bushes.

Since truly nested types are a special case of deep nested types, our methodology can derive useful induction rules for them — including the perpetually problematic truly nested type of bushes [8,10,15] introduced in Section 3.

*Example 5.* Since the truly nested type $Bush\,\alpha := \big(\mu\varphi^1.\lambda\beta.\,1 + \beta \times \varphi\,(\varphi\,\beta)\big)\,\alpha \in \mathcal{N}^\alpha$ is uniform in its index $\alpha$, it induces a type constructor $Bush := \mu\varphi^1.\lambda\beta.\,1 + \beta \times \varphi\,(\varphi\,\beta)$. Writing $H$ for $H_{[]}$ and $\hat{H}$ for $\hat{H}_{[]}$, and letting

$$H\;F\;A \;=\; [\![1 + \beta \times \varphi\,(\varphi\,\beta)]\!]^{\mathsf{Set}}\sigma[\beta := A][\varphi := F] \;=\; 1 + A \times F(FA)$$

we have that $\mu H = Bush$ and the predicate counterpart $\hat{H}$ to $H$ is given by

$$\begin{aligned}\hat{H}\,(F,P)\,(A,Q) &= [\![1 + \beta \times \varphi\,(\varphi\,\beta)]\!]^{\mathsf{Fam}}\overline{\sigma}[\beta := (A,Q)][\varphi := (F,P)]\\ &= (1,K_1)\,\underline{+}\,(A,Q)\,\underline{\times}\,(F,P)((F,P)(A,Q))\\ &= (1 + A \times F(FA),\ K_1 + Q \times \pi_2((F,P)((F,P)(A,Q))))\end{aligned}$$

Reflecting $\mu\hat{H}$ back into syntax gives the inductive predicate

$$\begin{aligned}
&\texttt{Bush}^\wedge : \forall(\mathsf{a} : \mathsf{Set}) \to (\mathsf{a} \to \mathsf{Set}) \to (\texttt{Bush}\,\mathsf{a} \to \mathsf{Set})\ \texttt{where}\\
&\quad \texttt{BNil}^\wedge : \forall(\mathsf{a} : \mathsf{Set})\,(\mathsf{Q} : \mathsf{a} \to \mathsf{Set}) \ \to \texttt{Bush}^\wedge\,\mathsf{a}\,\mathsf{Q}\,\texttt{BNil}\\
&\quad \texttt{BCons}^\wedge : \forall(\mathsf{a} : \mathsf{Set})\,(\mathsf{Q} : \mathsf{a} \to \mathsf{Set})\,(\mathsf{x} : \mathsf{a})\,(\mathsf{y} : \texttt{Bush}\,(\texttt{Bush}\,\mathsf{a})) \to\\
&\qquad\qquad \mathsf{Q}\,\mathsf{x} \to \texttt{Bush}^\wedge\,(\texttt{Bush}\,\mathsf{a})\,(\texttt{Bush}^\wedge\,\mathsf{Q})\,\mathsf{x} \to \texttt{Bush}^\wedge\,\mathsf{a}\,\mathsf{Q}\,(\texttt{BCons}\,\mathsf{x}\,\mathsf{y})
\end{aligned}$$

Now, if $P$ is any other predicate on *Bush* admitting an $\hat{H}$-algebra structure, then there must exist a morphism

$$\begin{aligned}
k :\ & \forall(x : 1 + \mathit{Bush}\,(\mathit{Bush}\,A)).\\
&\qquad (K_1 + Q \times \pi_2((\mathit{Bush}, \hat{P})((\mathit{Bush}, \hat{P})(A, Q))))x \to P\,A\,Q\,(\mathit{in}\,x)\\
&= \forall(x : 1 + \mathit{Bush}\,(\mathit{Bush}\,A)).\,(K_1 + Q \times P\,(\mathit{Bush}\,A)\,(P\,A\,Q))x \to P\,A\,Q\,(\mathit{in}\,x)
\end{aligned}$$

i.e., $(k_1, k_2)$, where $k_1 : \forall(x : 1).\,1 \to P\,A\,Q\,\mathit{BNil}$ and $k_2 : \forall(x : A).\ \forall(y : \mathit{Bush}\,(\mathit{Bush}\,A)).\,1 \to P\,(\mathit{Bush}\,A)\,(P\,A\,Q)\,y \to P\,A\,Q(\mathit{BCons}\,x\,y)$. Since `Bush^` reflects the initial $\hat{H}$-algebra, there is a unique predicate morphism from $\mu\hat{H}$ to $P$. Reflecting this morphism back into syntax gives the deep induction rule for bushes from Section 3.

The function `BDind`⇒`MBDind` in our Agda code shows that our methodology also derives a mutually recursive deep induction rule for bushes, there called `MBDind`.

Examples 4 and 5 show that when the definition of a nested type contains an instance of *another* nested type constructor `C` — e.g., `Maybe a` in the argument `Lam (Maybe a)` to `Abs` — its inductive predicate definition, and thus its deep induction rule, will involve a call to the predicate interpretation `C^` of `C`. When the definition contains an instance of the constructor for *the same* type being defined — e.g., `Bush a` in the type argument `Bush (Bush a)` to `BCons` — its inductive predicate definition, and thus its deep induction rule, will involve a recursive call to the inductive predicate being defined. The treatment of a truly nested type is thus exactly the same as the treatment of any other nested type.

Independently of deriving induction rules, even defining some nested types in Agda requires turning off its termination checks in a few tightly compartmentalized places. For example, neither Coq nor Agda currently allows the definition of the bush data type because of the non-positive occurrence of `Bush` in the type of `BCons`. The correctness of our development in those places is justified by [13]. This work suggests that the current notion of positivity should be generalized.

### 6 Related Work and Directions for Further Investigation

As far as we know, the phenomenon of deep induction has not previously even been identified, let alone studied. This paper treats deep induction for *nested types*, which extend ADTs by allowing higher-order recursion. Other generalizations of ADTs are also well-studied in the literature, including *(indexed) containers* [1,2], which extend ADTs by allowing type dependency. In particular, [3] defines a class of "nested" containers corresponding to inductive types whose constructors can recursively depend on the data type at different instances than the one being defined. The case of *truly* nested types is not treated there, however. We hope eventually to extend the results of this paper to derive provably correct deep induction rules for (indexed) containers, GADTs, dependent types, and other classes of more advanced data types. One interesting question is whether or not a common generalization of indexed containers and the class of nested types studied here has a rigorous initial algebra semantics as in [13].

A more recent line of investigation concerns *sized types* [5]. These are particularly well-suited to termination checking of (co)recursive definitions, and are implemented in the latest versions of Agda [6]. Although originally defined in the context of a type theory with higher-order functions [4], the current incarnation of sized types does not appear to admit definitions with true nesting. What seems to be missing is an addition operation on sizes, which would allow a constructor such as `BCons` to combine a structure with size of depth "up to $\alpha$" with one of depth "up to $\beta$" to define a data element of depth "up to $\alpha + \beta$".

Tassi [17] has independently implemented a tool for deriving induction principles of data type definitions in Coq using unary parametricity. Although neither rigorous derivation nor justification is provided, his technique seems to be essentially equivalent to ours, and could perhaps be justified by our general framework. True nesting still is not permitted, however. In [7], mutually recursively defined induction and coinduction rules are derived for mutually recursive and corecursive data types. But these are still the standard structural (co)induction rules, rather than deep ones. This suggests a need for deep coinduction rules, too.
